# Maternal education and child immunization: the mediating roles of maternal literacy and socioeconomic status

**DOI:** 10.11604/pamj.2017.26.217.11856

**Published:** 2017-04-24

**Authors:** Saliu Adejumobi Balogun, Hakeem Abiola Yusuff, Kehinde Quasim Yusuf, Abdulah Mohammed Al-Shenqiti, Mariam Temitope Balogun, Prudence Tettey

**Affiliations:** 1 Menzies Institute for Medical Research, University of Tasmania, Australia; 2 Otun Centre for Health and Social Research, Lagos, Nigeria; 3 State Ministry of Health, Abeokuta, Ogun State, Nigeria; 4 College of Medical Rehabilitation Sciences, Taibah University, Madinah, Saudi Arabia; 5 Life without Barriers, Tasmania, Australia

**Keywords:** Immunization, education, literacy, socioeconomic status, mediation, Nigeria

## Abstract

**Introduction:**

Previous studies in Nigeria have documented significant association between maternal education and child immunization. However, little is known about the pathway through which maternal education improves immunization uptake. This study aims to examine whether maternal literacy and socioeconomic status mediates the relationship between maternal education and complete immunization coverage in children.

**Methods:**

Nationally representative data from the first wave of the Nigeria General Household Survey-Panel were used, which includes 661 children aged one year and below. Regression analyses were used to model the association between maternal education and child's immunization uptake; we then examined whether maternal literacy and household economic status mediates this association.

**Results:**

Of the 661 children, 40% had complete immunization. The prevalence ratio (PR) of complete immunization in children whose mothers were educated versus those whose mothers were not educated was 1.44 (95% CI: 1.16-1.77). Maternal literacy substantially reduced the estimated association between maternal education and complete immunization by 90%, whereas household economic status reduced the estimates by 27%.

**Conclusion:**

These findings suggest that complete immunization was higher in children whose mothers were educated, partly because maternal education leads to acquisition of literacy skills and better health-seeking behavior which then improves immunization uptake for their children. Socioeconomic status is an alternative pathway but with less substantial indirect effect.

## Introduction

Full course of vaccinations against debilitating diseases such as poliomyelitis, tuberculosis, measles, diphtheria, and neonatal tetanus is one of the most cost-effective public health strategies to reduce child morbidity and mortality [1, 2]. In addition to reducing the risk of the diseases for which they are intended, vaccines such as Bacillus Calmette-Guérin (BCG) and measles-containing vaccines (MCV) also lower the risk of illness and mortality from other causes [3]. In spite of these benefits, there is significant variability in vaccination coverage in different regions of the world with lower coverage among children in low and middle income countries, including sub-Sahara Africa [4]. For instance, in 2015, the ten countries with the lowest coverage of the third dose of Diphtheria-Pertussis-Tetanus containing vaccine (DPT3) are in low income countries and seven of these countries are in Africa [4]. The World Health Organization Expanded Programme on Immunization (EPI) was initiated in Nigeria in 1979; since then considerable efforts has been made towards ensuring universal immunization coverage in the country [1, 2, 5]. Despite these efforts, inequality in access to immunization has been documented. For instance, several studies in Nigeria like other sub-Saharan African countries have provided evidence that maternal education is associated with reduced risk of incomplete immunization as well as reduced risk of other child health outcomes such as malaria, malnutrition and mortality [6-8]. This evidence was documented in a Nigerian study which reported that the odds of complete immunization was 3.6 times higher in children whose mothers had at least a secondary school education compared to those with lower or no education [7]. In spite of the extensive study on maternal education and childhood immunization in Nigeria, limited studies have examined the pathway through which maternal education improves immunization. Identifying factors that inhibit uneducated mothers from completing immunization for their children is crucial for designing interventions to improve immunization uptake in this sub-group of mothers. Prior studies suggest that maternal reading skills partly explain the association between maternal education and infant mortality [8]. It is unclear whether the relationship between maternal education and complete immunization follows a similar pathway. Using a nationally representative data, this study aims to examine the relationship between maternal education and immunization in children younger than 12 months. We also aim to examine whether maternal literacy and socioeconomic status mediate the relationship between maternal education and child immunization.

## Methods

Data for children one year and below were analysed from the Nigeria General Household Survey-Panel (Nigeria GHS-Panel). The Nigeria GHS-Panel is a nationally representative household survey conducted by the Nigerian National Bureau of Statistics, with support from the World Bank [9]. The survey provides a reliable estimate of key socio-economic variables for the six geopolitical zones in Nigeria [9]. The Nigeria GHS-Panel was carried out twice in each wave, once after the planting season (post-planting visit), and the other after the harvest season (post-harvest visit). Data collected from both the post-planting (August-October, 2010) and post-harvest (February- April 2011) visits were used in this study. Response rates at the first (post-planting) and second (post-harvest) visits were 99.7% and 97.0% respectively, constituting excellent participation rates, and therefore supporting the robustness of the data. The Nigeria GHS-Panel survey used a multi-stage sampling technique that randomly selected 5,000 households, involving 27,533 household members. A total of 661 children aged one or below were included in the analysis.

**Outcome measure: complete immunization**: The outcome measure used in this analysis was complete immunization. A child is considered to be completely vaccinated if he or she has received a dose of MCV, BCG, three doses of DPT and three doses of oral polio vaccine (OPV) [5]. Immunization status for each vaccine was assessed based on response to the question asking whether or not the child has the vaccination.

**Explanatory variables**: Maternal education was assessed based on a response to the question asking whether the participants have ever attended school. Response to this question was dichotomised (0 = attended school, 1= never attended school). Household economic status was measured as tertiles of per capita household expenditure (that is, total household expenditure divided by the household size) [10]. Household expenditure represents the total expenses paid for food and non-food items (i.e. health, housing, electricity and other goods and services) in each household. The household expenditure data was collected twice in the survey (during the post-planting and post-harvest visits); hence, the aggregated household expenditure was the average household expenditure for both visits combined. Household expenditure as captured in this paper is a direct measure, and is, therefore, the preferred measure for living standards, as the collection of expenditure data is more reliable than utilising a measure such as income. Maternal literacy was assessed based on response to the question asking whether a participant could read and write in any language. Response to this question was dichotomised (0 = no, 1= yes). Other explanatory variables included in the analysis are maternal age, child's gender and age.

**Figure 1:**
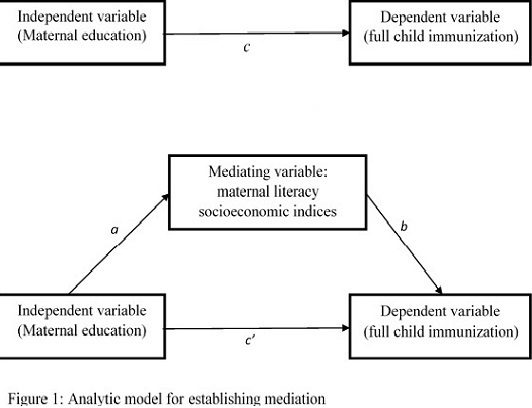
Analytic model for establishing mediation

*Data analysis*: descriptive statistics (Pearson chi-square tests) for each explanatory variable were compared for children who completed complete immunization and those who do not. Generalised linear model with robust standard error was used to estimate the prevalent ratios (PR) of complete immunization [11]. To establish whether maternal literacy and household economic status mediate the relationship between maternal education and complete immunization, series of regression analyses were performed as suggested by Baron and Kenny [12]. First, we performed a regression of the dependent variable (complete immunization) on the independent variable (maternal education) (Figure 1, path c). Next we perform a regression of the mediator (maternal literacy and household economic status) on the independent variable (maternal education) (Figure 1, path a). We also established a relationship between the mediating variables and the dependent variable by performing a regression of the dependent variable (complete immunization) on the mediating variable (maternal literacy and household economic status) (Figure 1, path b).

The purpose of the analyses in paths a, b and c is to establish that there is a significant relationship among the variables. In the final model, we perform a regression of the dependent variable on both the mediator and the independent variable (Figure 1, path c'). In this final model, the magnitude of effect of maternal education on complete immunization (in path c') should be smaller than the estimates in path c, since the proportion mediated is accounted for by the mediating variable. The mediating role of household economic status and maternal literacy in the relationship between maternal education and complete immunization in children is established if all of the criteria described above are met. We assessed the statistical significance of the mediation effect using the binary mediation command in Stata [13]. The binary mediation command computes the indirect effect of a mediating variable using standardized coefficients, this approach has been previously suggested for mediation analysis involving binary variables [13]. For ease of interpretation we reported prevalence ratio in the main text and the estimated standardized coefficients are provided in supplementary table. Confidence interval and statistical test of significance for the proportion mediated were estimated using bootstrapping with 500 replications. Evidence for a possible exposure-mediator interaction was assessed by a test of significance of a (maternal education × mediators) product term. We evaluated multi-collinearity among all the predictor variables to identify possible collinearity bias. There was no variance inflation factor of greater than 10 suggesting no evidence for multi-collinearity [14]. Data analysis was performed using Stata version 13 (StataCorp, TX, USA), and P<0.05 was considered statistically significant.

## Results

Table 1 presents descriptive characteristics of the participants stratified by child's immunization status. There was no statistical significant difference in age of mothers whose children completed immunization (mean ± SD: 28.9 ± 6.2) and those who do not (mean ± SD: 28.5 ± 6.9). Complete immunization was lower in male children (34.6% vs. 45.4% in female children) and in children whose mothers were not educated (31.3% vs. 45.0% in children of educated mothers) and in illiterate mothers (30.5% vs. 48.9% in children of literate mothers). Complete immunization was also lower in children living in poor households (lowest tertile: 31.8%, middle: 39.0% and highest tertile: 49.3%). Table 2 shows the association between the maternal education and complete immunization in children. In the unadjusted model (Table 2, column 2), prevalence ratios (PR) of complete immunization was higher in children whose mothers were educated (PR=1.44, 95% CI: 1.16-1.77). The higher PR remained after adjusting for child's gender and age (PR=1.44, 95% CI: 1.16-1.78) (Table 2, column 3). After adjusting for maternal literacy (Table 2, column 4), the association between maternal education and complete immunization in children becomes non-significant (PR=1.04, 95% CI: 0.77-1.40, P=0.808) and the standardized magnitude of the effect of maternal education on complete immunization was reduced by 90% (P-value for mediation =0.001). The PR of complete immunization was lower after controlling for household economic status (PR=1.29, 95% CI: 1.04-1.60, P=0.022), and the standardized magnitude of the effect of maternal education on complete immunization in children was reduced by 27%. The statistical test of significance for the proportion mediated by household economic status was significant (P=0.008). There was no evidence for exposure-mediator interaction between maternal education and maternal literacy (P=0.628), and household economic status (P=0.983).

**Table 1:** descriptive statistics of explanatory variables stratified by child’s immunization status

Variables	Total	Incomplete immunization		Complete immunization		*P*-value
		n	% or mean(SD)	n	% or mean(SD)	
**Maternal age (Mean (SD))**	652	393	28.5 (6.9)	259	28.9 (6.2)	0.520
**Maternal education**						
Non-educated	255	175	68.6	80	31.3	0.001
Educated	393	216	55.0	177	45.0	
**Maternal literacy**						
Illiterate	325226		69.5	99	30.5	<0.001
Literate	325166		51.1	159	48.9	
**Child’s sex**						
Male	346	226	65.3	120	34.6	0.005
Female	206	167	54.6	139	45.4	
**Child’s age**						
Less than 12 months	371	132	64.4	239	35.6	0.013
12 months old	281	127	54.8	154	45.2	
**Household economic status‡**						
Lowest	221	144	68.3	67	31.8	
Second	223	136	61.0	87	39.0	0.001
Highest	199	101	50.8	98	49.3	

‡ Tertiles of per capita household expenditure

**Table 2:** Prevalent ratios (PR) of complete immunization by children aged one and below in Nigeria

	Unadjusted PR (95% CI)	Model 1 PR (95% CI)	Model 2 PR (95% CI)	Model 3 PR (95% CI)
**Maternal education**				
Not educated mothers	1.00	1.00	1.00	1.00
Educated mothers	1.44 (1.16-1.77)	1.44 (1.16-1.78)	1.04 (0.77-1.40)	1.29 (1.04-1.60)
*Model sample size*	648	648	648	629
*Log pseudolikelihood*	– 490.92	– 486.93	– 483.85	– 469.26

* Data in bold indicates statistical significance at *P* < 0.05; Model 1: adjusted for child’s age and sex; Model 2: Model 1 + maternal literacy; Model 3: Model 1 + household economic status

## Discussion

A major step to ensure equitable access to immunization is to understand why some children are not fully immunized. Using a nationally representative data, this study aims to examine the pathway through which maternal education improves immunization in Nigerian children. We found that complete immunization uptake was higher in children whose mothers were educated and that maternal literacy substantially reduced the magnitude of the effect of maternal education on complete immunization in children. Higher socioeconomic status is another pathway through which maternal education improves immunization uptake in children. However, the indirect effect of socioeconomic status is lower compared to maternal literacy. Maternal literacy significantly reduced the magnitude of effect of maternal education on child immunization by 90%, making the relationship non-significant. This finding provides evidence for the mediating role of maternal literacy in the relationship between maternal education and child immunization. Although, education may lead to an improvement in health knowledge, greater health knowledge may not be a direct consequence of the curriculum covered in school, but a consequence of academic skills, particularly literacy skills which may help mothers to become receptive to health information via sources such as mass media [15, 16]. This finding is consistent with a prior study in Nigeria which considered child mortality outcome. Using data from the 2003 Nigerian Demographic and Health Survey, Smith-Greenaway showed that maternal reading skills mediate the relationship between maternal education and child mortality [8].

Our finding has implication for interventions aimed at improving complete immunization among children in Nigeria, particularly among those whose mothers were not educated as it suggests literacy as a pathway through which education improves immunization uptake. Reading skills among women of reproductive age in Nigeria has been shown to increase linearly with years of schooling; however, there are many women with several years of education who are unable to read at all [8]. Hence, strategies to improve complete immunization uptake should address effective and quality education that would improve maternal reading or literacy skills and not just improving female school enrolment or enrolment into adult education programmes. Household economic status accounts for 27% of the total effects of maternal education on complete immunization in children. This finding suggests that socioeconomic status partly explain why complete immunization was higher in children whose mothers were educated. Prior studies have provided evidence that higher levels of education improves income and socioeconomic status [17] and that children of parents in higher socioeconomic status are more likely to complete immunization [18]. Routine immunization is free for children in Nigeria, hence, the low immunization uptake in mothers in low socioeconomic status may not be wholly related to reduced financial capacity to pay for immunization but could be a reflection of some forms of disadvantage of mothers in low socioeconomic status. This strength of this study includes a very high response rate and the use of a nationally representative dataset, which increases the generalizability of our findings. The study has some limitations. First, child immunization status was self-reported and may be subjected to recall-bias; however, prior studies have shown similarity between other indicators of vaccination with maternal recall [19]. Secondly, we assessed maternal socioeconomic status using household aggregated data which may not reflect the mother's access to material resources. Lastly, the cross-sectional nature of the study precludes ascription of causality. Despite these limitations, this study is crucial as it contributes to understanding why immunisation uptake is lower in children whose mothers were not educated.

## Conclusion

In conclusion, our finding indicates that the relationship between maternal education and immunization uptake could be substantially explained by maternal literacy. Household economic status is another pathways through which maternal education improves immunization uptake. However, the indirect effect of these socioeconomic status is lower compared to maternal literacy skills.

### What is known about this topic

- Children of mothers who are educated are likely to complete their immunization;
- Limited studies have examined the pathway through which maternal education increase immunization uptake.

### What this study adds

- Mothers who were educated were able to complete immunization for their children because maternal education leads to acquisition of literacy skills and better health-seeking behavior which then improves immunization uptake for their children;
- Higher socioeconomic status is another pathway (but with less substantial indirect effects compared to maternal literacy) through which maternal education improves immunization uptake in children.
